# Slow evolution under purifying selection in the gamete recognition protein bindin of the sea urchin *Diadema*

**DOI:** 10.1038/s41598-020-66390-2

**Published:** 2020-06-17

**Authors:** L. B. Geyer, K. S. Zigler, S. Tiozzo, H. A. Lessios

**Affiliations:** 1 Smithsonian Tropical Research Institute, Apartado Postal 0843-03092, Balboa, Ancon Panama; 2 Department of Biology, Sewanee: University of the South, 735 University Ave., Sewanee, TN 37383 United States; 3 Sorbonne Universite, CNRS, Laboratoire de Biologie du Developpement de Villefranche-sur-mer (LBDV), 06230 Paris, France

**Keywords:** Molecular evolution, Sexual selection, Speciation

## Abstract

Bindin is a sperm protein that mediates attachment and membrane fusion of gametes. The mode of bindin evolution varies across sea urchin genera studied to date. In three genera it evolves under positive selection, in four under mostly purifying selection, and in one, results have been mixed. We studied bindin evolution in the pantropical sea urchin *Diadema*, which split from other studied genera 250 million years ago. We found that *Diadema* bindin is structurally similar to that of other genera, but much longer (418 amino acids). In seven species of *Diadema*, bindin evolves under purifying selection, more slowly than in any other sea urchin genus. Only bindin of the recently rediscovered *D. clarki* shows evidence of positive selection. As *D. clarki* is sympatric with *D. setosum* and *D. savignyi*, positive selection could arise from avoidance of maladaptive hybridization. However, *D. setosum* and *D. savignyi* overlap in the Indo-West Pacific, yet their bindins show no evidence of positive selection, possibly because the two species spawn at different times. Bindin in the East Pacific *D. mexicanum*, the West Atlantic *D. antillarum*, the East Atlantic *D. africanum*, and the Indo-Pacific *D. paucispinum* also evolves slowly under purifying selection.

## Introduction

Many marine organisms reproduce by free-spawning gametes into the water column. Interactions between sperm and egg, mediated by molecules that affect species recognition and fertilization, are of particular importance in this mating system. The evolution of two of these molecules, bindin in sea urchins and lysins in gastropods and bivalves, has been studied most intensively (reviews in refs. ^1–6^). Gamete recognition proteins (GRPs) are often thought to evolve under strong positive selection^1,7,8^; the evolution of lysins conforms to these expectations. Evolution in bindins, on the other hand, is much more varied and is different from one genus to the next. Although, in general, divergence in bindin is correlated to the degree of gamete compatibility between species^9^, it is not one of the fastest evolving molecules of sea urchins^10^. Among the genera in which it has been studied thus far, bindin shows evidence of positive selection in three, *Echinometra*^11–14^, *Strongylocentrotus (sensu lato)*^15,16^, and *Paracentrotus*^17^, but there is no such evidence in four, *Arbacia*^18,19^, *Lytechinus*^20^. *Pseudoboletia*^21^, and *Tripneustes*^22^. In an additional genus, *Heliocidaris*, results are mixed, and depend on the species that are included in the analysis^23–25^. Various reasons for these differences have been postulated, but there is no single explanation that fits all genera^1,2,26^. Reinforcement^1,14,18^, male-female conflict^27^, sperm competition^28^, and assortative mating^29,30^, have all been proposed as possible sources of selection driving this evolution. It has become obvious that generalizations made in the early stages of the study of bindin evolution no longer apply to the entire class Echinoidea, and that different selective forces are most likely acting on bindins in different species. Measuring fertilization success of different bindin alleles has produced valuable insights in three species, *Echinometra mathaei*^30^, *Mesocentrotus* (formerly *Strongylocentrotus) franciscanus*^27^, and *S. purpuratus*^31^. The generality of the findings in these three species for the entire class, however, remains in question. It is, thus, important to document patterns of evolution in as many taxa of sea urchins as possible. The present study deals with the evolution of bindin in the sea urchin *Diadema*, a genus that occurs in tropical seas of all oceans. *Diadema* belongs to the order Diadematoida, the bindin of which, except for a single sequence in a survey of bindins of the class Echinoidea^32^, has not been studied.

The genus *Diadema* contains both sympatric and allopatric species^33^. *D. antillarum* occurs in the tropics and subtropics of the western Atlantic, *D. africanum* in the eastern Atlantic^34^, and *D. mexicanum* in the eastern Pacific; *D. savignyi* and *D. setosum* co-occur in the western Pacific and the Indian Ocean, but not in the central Pacific, where only *D. savignyi* is present^35^; *D. palmeri* is endemic to New Zealand and the eastern and southern coasts of Australia^36^. The range of *D. paucispinum* was thought to be restricted to Hawaii and Johnson Island, but Lessios *et al*.^33^ found that its mitochondrial DNA spreads all the way to the Indian Ocean. *D. clarki* has been recently rediscovered in Japan^37,38^ and Indonesia^39^, but may also be present in other areas, such as the Marshal Islands^33^. Gametes of *D. setosum* and *D. savignyi* are capable of fertilizing each other in the laboratory and of producing viable hybrids^40^. Isozymes of morphologically intermediate individuals showed that natural hybrids of *D. setosum, D. savignyi*, and *D. paucispinum* do occur, but that introgression is low^41^.

*Diadema* exhibits behavioral mechanisms which promote fertilization success, including spawning aggregations^42–44^ and intraspecifically synchronized lunar spawning cycles^45–49^. *D. savignyi* spawns at the full moon, whereas the partially sympatric *D. setosum* spawns at the new moon^48^. *D. mexicanum* and *D. antillarum*, separated by the Isthmus of Panama, also spawn at different phases of the moon^45^.

A mitochondrial phylogeny by Lessios *et al*.^33^ showed that there are two deeply divided clades in *Diadema*, one that leads to two presumed separate species that are formally still recognized as *D. setosum*, and another that includes all other species of the genus. *D. savignyi, D. antillarum*, *D. africanum* and *D. paucispinum* are members of a polytomy, while *D. mexicanum* is a sister clade to this polytomy. *D. clarki* split from these earlier, and *D. palmeri* earlier still, shortly after the split from *D. setosum*.

We sequenced bindin genes from all the described and suspected species of the genus *Diadema* to examine the mode of evolution of this gene. We asked whether this protein evolves under positive selection, as it does in three other sea urchin genera, or by negative selection, as it does in another four, and whether patterns of sympatry and allopatry have affected the mode of bindin evolution of particular species.

## Methods

Tissue samples preserved in high salt DMSO buffer^50^ or 95% ethanol were collected from multiple populations of all eight described species of *Diadema* by various collectors (see Acknowledgements). Total DNA was extracted by Proteinase K digestion as described in Lessios *et al*.^51^.

The sequence of the *Diadema antillarum* bindin mRNA precursor^32^ (GenBank Accession AY126485.1), was used to design primers for amplifying either the full length mature bindin including the intron (primers DA5A-DA3R2, amplified length 1986–2010 bp), or the first exon from the prepro bindin region up to the intron/exon boundary (primers DA5A-DAIR, amplified length 677–680 bp) (Supplementary Table S1). Forty cycles of Polymerase chain reaction (PCR) amplification (94 °C for 45 s, 50–55 °C for 30 s, and 72 °C for 30 s, followed by incubation at 72 °C for 5 minutes) were carried out on each sample using DyNazyme polymerase (Finnzyme) according to the manufacturer’s recommendations. Amplicons were cloned using TA-cloning (pGEM-T Easy Vector System, Promega). A minimum of 5 clones per individual were sequenced by cycle-sequencing (BigDye Terminator v3.1, Applied Biosystems) on a 3130 Genetic Analyzer (Applied Biosystems), using standard vector primers and the internal sequencing primers shown in Supplementary Table S1. Consensus sequences of 2–3 clones per allele were constructed to reduce cloning errors. Additional clones were sequenced on an ad-hoc basis to eliminate suspected errors and ambiguities wherever necessary. Bindin from a single individual of the diadematid *Echinothrix diadema* collected at Isla del Coco, Costa Rica was sequenced by the same methods to serve as an outgroup.

We obtained sequences of the first exon of bindin from all currently recognized or suspected species of *Diadema*. We also obtained full length bindin sequence from a subset of individuals of *D. antillarum*, *D. savignyi*, *D. paucispinum*, *D. mexicanum, D. africanum* and *D. clarki*. Despite multiple attempts, we were unable to amplify the full length bindin sequence of *Diadema setosum* or of *D. palmeri;* analyses including these two species are limited to the first exon. Sequences were submitted to GenBank under Accession Numbers MT365802-MT365868 and MT375187- MT375188.

Allelic sequences were aligned by eye using Sequencher 5.3 (Gene Codes). For phylogenetic analyses these alignments were further aligned to the outgroup sequence in MAFFT 7^52^ using the E-INS-i iterative refinement model. Phylogenetic trees were constructed using Maximum Likelihood (GARLI 0.951^53^) and Bayesian (MrBayes 3.2.6^54^) methods. For the maximum likelihood analysis, 500 bootstrapped replicate runs were performed, using a generalized GTR model^55^, estimating all parameters. The Bayesian analysis was performed using a 4 × 4 DNA substitution model with equal variation across sites. A flat Dirichlet prior was used for the substitution model, with a beta distributed prior for the transition/transversion ratio. The analysis was run for 50,000,000 steps, sampling every 1000, using 2 runs and 4 chains. One quarter of the initial values were discarded as burnin. A maximum value of 0.01 in the standard deviation of the split frequencies was used as an indication that the chains had converged.

Kimura 2-parameter distances^56^ were calculated using MEGA 7.0.25^57^. The proportions of silent (d_S_) and amino acid replacement (d_N_) mutations were calculated according to the methods of Pamilo and Bianchi^58^ and Li^59^. Codon based Z-tests of departure from selective neutrality were performed using the method of Nei and Gojobori^60^.

Two alignments, one of the first exon of bindin that included all species, and a second of the full length mature bindin molecule, which excluded those species for which we were unable to obtain second exon sequence, were each tested separately for recombination using the Genetic Algorithm Recombination Detection (GARD)^61^ program as implemented in HyPhy on the Datamonkey server^62,63^, using two rate classes and an optimized substitution model chosen by the Datamonkey Model Selection tool.

Maximum Likelihood analyses for positive selection were carried out using the codeml module of PAML 4.8^64,65^, which uses the method of Yang *et al*.^66^ to model changes in the ratio of non-synonymous to synonymous nucleotide changes (ω) among sites. Because recombination was detected between the first and second bindin exon, which, if not taken into consideration, can cause false positive results, and because we were unable to obtain second exon sequences of *D. setosum* and *D. palmeri*, separate analyses were carried out on the first and second exons of bindin. A neighbor-joining^67^ unrooted bifurcating tree was constructed in Paup* 4.0a (build 159)^68^ to serve as the basis for each PAML analysis. These trees were largely compatible with the topology of the Bayesian and Maximum Likelihood trees, with only small differences in the arrangements of the terminal nodes. We used likelihood ratio tests to compare three sets of standard sites models to evaluate the possibility of positive selection: M1a (nearly neutral) vs. M2a (positive selection)^69,70^, M7 (Beta) vs. M8 (Beta plus ω)^66^, and M8a vs. M8^69,71^. The M7/M8 comparison is slightly less conservative than the M1a/M2a in comparing a model of positive selection to a neutral model. The M8a/M8 comparison has fewer parameters and, therefore, more statistical power than M7/M8, but may underestimate positive selection when the value of ω is close to 1. Two additional sets of models using the method of Bielawski and Yang^72^, implementing changes in ω across clades, rather than across sites, were also tested against the neutral model 2a_rel of Weadick and Chang^73^. In these models, three site classes are estimated for each branch, with the first class in each model constrained between 0 and 1. Model C requires one of the site classes to have ω fixed at 1, forcing a neutral class, whereas model D allows all classes to vary freely. For the two latter models, a clade formed by the bindin of *D. antillarum*, *D. africanum*, *D. paucispinum*, *D. savignyi*, and *D. mexicanum* served as the background, and (in the analysis of the first exon) the clades of the bindins of *D. clarki*, *D. palmeri*, and *D. setosum* were each allowed to vary individually, resulting in a model with five classes of ω. For the second exon, for which we lacked data from *D. palmeri* and *D. setosum*, there were two classes of ω, with the same set of background branches and only *D. clarki* serving as the foreground clade.

Additional analyses, for both positive and negative selection at individual amino acid sites, were carried out in HyPhy as implemented on Datamonkey. The DNA segments between breakpoints and trees estimated by GARD were tested in Single Likelihood Ancestor Counting (SLAC)^63^, Fixed Effects Likelihood (FEL)^63^, Mixed Effects Model of Evolution (MEME)^74^, and Fast Unconstrained Bayesian Approximation (FUBAR)^75^ methods.

## Results

### Structure of the *Diadema* bindin gene

The mature bindin molecule (Supplementary Fig. S1 & S2) of *Diadema* is composed of 418 amino acid residues, 130–212 more than that of other sea urchin genera studied to date^32^. *Diadema* bindin shares with the bindin of all other sea urchin genera studied to date a 55 amino acid conserved core and an intron inserted after a conserved Valine^32^. In *Diadema* this intron is 481 to 505 bp long. Like *Arbacia*, *Eucidaris, Heliocidaris*, and *Encope*, the bindin of *Diadema* lacks the Glycine-rich repeat regions found in bindins of *Echinometra*, *Strongylocentrotus (sensu lato), Lytechinus, Paracentrotus, Tripneustes, Pseudoboletia*, and *Moira* and, thus, the associated length variation seen in some of these genera^11–13,15–17,20,21,23^. Repetitive sequences are often associated with the generation of length mutations^76^ and with recombination hotspots^77^. In *Diadema* there were two single codon indels in the first exon and five larger indels on the second exon, but these were irregularly spaced compared to the large indels associated with repeats in the bindins of other genera. Five out of seven observed indels in the entire alignment were unique to *D. clarki*. GARD indicated a high probability of recombination across the intron but not within either exon. Relatively high recombination rates within the exons has been observed in the bindin molecules of some of the other genera^14,20,78^.

### Genealogy of *Diadema* Bindin

**Figure 1:**
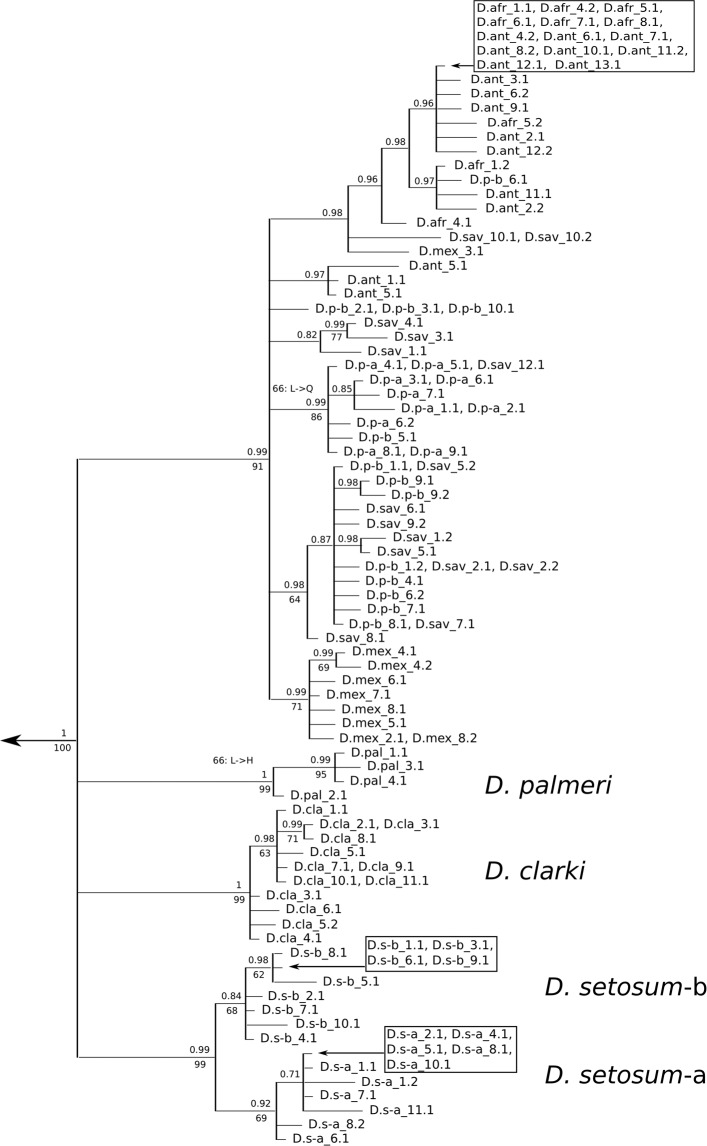
Gene genealogy of *Diadema* bindin first exon alleles. The tree was constructed using MrBayes and rooted on an allele of *Echinothrix diadema*. Numbers above the branches indicate Bayesian confidence estimates. Branches with less than 0.70 support were collapsed. Numbers below the branches indicate bootstrap support from GARLI maximum likelihood analysis; bootstrap support of less than 60% is not shown. Numbers on the terminal branches indicate the individual from which an allele came, then, after the period, the identity of the allele. For homozygous individuals, only one allele is shown. Species codes: D.afr: *Diadema africanum*, D. ant: *D. antillarum*, D.p-a: *D. paucispinum*-a (as in Lessios *et al*.^33^), D.p-b: *D.paucispinum*-b, D.sav: *D. savignyi*, D.mex: *D. mexicanum*, D.cla: *D. clarki*, D.pal: *D. palmeri*, D.s-a: *D. setosum*-a (as in Lessios *et al*.^33^), D.s-b: *D. setosum*-b. Transitions between amino acids identified by MEME as being under positive selection are marked along the branches by showing the amino acid positions in their alignment (Supplementary Fig. S1), and the identity of the amino acids.

Reconstruction of the genealogy of bindin alleles in *Diadema* using maximum likelihood and Bayesian methods converged on similar topologies, differing mainly in the details of the terminal nodes, which had low support. Figure 1 presents the Bayesian tree of the first bindin exon. Bindin first exons in *D. antillarum, D. africanum, D. paucispinum*, and *D. savignyi*, species that in the mitochondrial phylogeny formed a polytomy^33^, were not monophyletic. Identical alleles were found in multiple species, including one which is shared between one individual of *D. paucispinum*-b and two individuals of *D. savignyi*, two which are shared by one individual of *D. paucispinum*-b and one of *D. savignyi*, one which is shared between two individuals of *D. paucispinum*-a and one *D. savignyi*, and one at relatively high frequency in both *D. antillarum* and *D. africanum*. One *D. mexicanum* allele is also found in this clade, but the rest form a monophyletic group. Only the most distantly related species (from each other and from the mitochondrial polytomy of the other species), *D. setosum*-a, *D. setosum*-b, *D. palmeri*, and *D. clarki*, are monophyletic at bindin.

**Figure 2:**
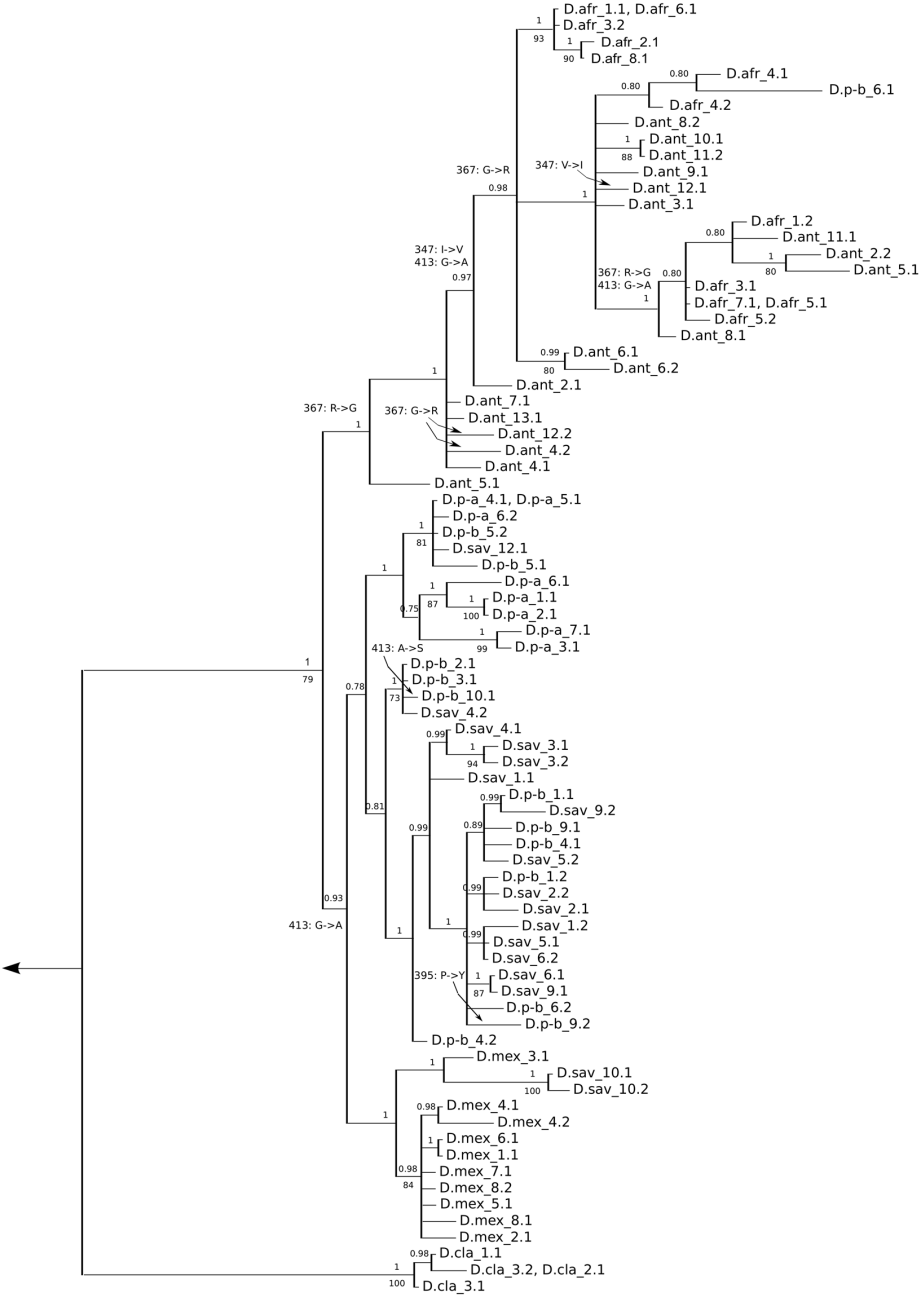
Gene genealogy of entire mature bindin alleles of *Diadema*. The tree was constructed using MrBayes and was rooted on an allele of *Echinothrix diadema*. Numbers above the branches indicate Bayesian confidence estimates. Branches with less than 0.70 support were collapsed. Numbers below the branches indicate bootstrap support from GARLI maximum likelihood analysis; bootstrap support of less than 60% is not shown. Numbers on the terminal branches indicate the individual from which an allele came, then, after the period, the identity of the allele. For homozygous individuals, only one allele is shown. Species codes: D.afr: *Diadema africanum*, D.ant: *D. antillarum*, D.p-a: *D. paucispinum*-a (as in Lessios *et al*.^33^), D.p-b: *D.paucispinum*-b, D.sav: *D. savignyi*, D.mex: *D. mexicanum*, D.cla: *D. clarki*. Transitions between amino acids about which FEL and FUBAR agree that they are under positive selection are marked along the branches by showing the amino acid position (Supplementary. S2), and the identity of the amino acids.

The genealogy of the longer sequence of the complete bindin gene of all species except *D. setosum* and *D. palmeri* (Fig. 2) added some resolution when compared to the analysis of the first exon of all species. It indicated a split between the bindins of the Atlantic species *D. antillarum* and *D. africanum*, on the one hand, and of the Indo-Pacific species *D. savignyi* and *D. paucispinum*, on the other, with the exception of a single sequence of *D. paucispinum*-b that falls within the Atlantic clade. The Bayesian phylogeny clustered bindin of *D. mexicanum* with the Indo-Pacific species, but the maximum likelihood reconstruction did not. Sub-clades within each of these clades were still polyphyletic.

### Analyses for the mode of selection

Calculations of amino acid replacement (d_N_) and silent (d_S_) substitutions of the first exon did not provide evidence of positive selection within species of *Diadema* (Table 1). In *D. clarki* and *D. palmeri* there were no silent substitutions, so that ω = ∞. In all other cases the ratio was less than 1 in all intraspecific comparisons. ω values of the entire bindin molecule in species where it could be sequenced were also <1, and not significantly different from the expectation of neutrality (Table 2). Comparisons between species also produced ω ratios <1 (Tables 3 & 4). The excess of silent mutations between species in the full mature bindin was significant in all cases after sequential Bonferroni corrections for multiple tests^79^, suggesting the presence of purifying selection.

**Table 1:** Number of individuals, number of alleles, mean Kimura 2-parameter distance, synonymous substitutions per synonymous site (d_S_), and nonsynonymous substitution per nonsynonymous site (d_N_) within species in the first exon (432 bp) of *Diadema* bindin.

Species	N	n alleles	K2	d_N_^a^	d_S_^a^	ω	
*D. africanum*	8	12	0.0027	0.0014	0.0046	0.3082	^ns^
*D. antillarum*	14	21	0.0079	0.0055	0.0165	0.3349	^ns^
*D. paucispinum-*a	9	10	0.003	0.0021	0.0073	0.2928	^ns^
*D. paucispinum-*b	10	15	0.0112	0.0046	0.0309	0.1484	^ns^
*D. savignyi*	11	19	0.0126	0.0056	0.0338	0.1647	^ns^
*D. mexicanum*	8	10	0.0069	0.0046	0.0133	0.3482	^ns^
*D. palmeri*	3	3	0.0016	0.0025	0.0000	∞	^ns^
*D. clarki*	11	13	0.0033	0.0048	0.0000	∞	^ns^
*D. setosum-*a	10	12	0.0035	0.0034	0.0068	0.5016	^ns^
*D. setosum-*b	10	10	0.0033	0.0014	0.0093	0.1469	^ns^

^a ^Pamilio and Bianchi^58^ and Li^59^ method.

^ns^ ω not significantly different from 1 after sequential Bonferroni correction^79^ for multiple tests.

**Table 2:** Number of individuals, number of alleles, mean Kimura 2-parameter distance, synonymous substitutions per synonymous site (d_S_), and nonsynonymous substitution per nonsynonymous site (d_N_) across the entire mature bindin gene.

Species	N	n alleles	K2	d_N_^a^	d_S_^a^	ω	
*D. africanum*	8	12	0.0093	0.0067	0.0163	0.4117	^ns^
*D. antillarum*	12	19	0.0118	0.0091	0.0196	0.4614	^ns^
*D. paucispinum* a	8	9	0.0070	0.0044	0.0145	0.3043	^ns^
*D. paucispinum* b	8	13	0.0096	0.0056	0.0234	0.2390	^ns^
*D. savignyi*	8	16	0.0089	0.0059	0.0194	0.3057	^ns^
*D. mexicanum*	8	10	0.0041	0.0030	0.0075	0.4009	^ns^
*D. clarki*	3	4	0.0020	0.0028	0.0000	∞	^ns^

^a ^Pamilio and Bianchi^58^ and Li^59^ method.

^ns^ ω not significantly different from 1 after sequential Bonferroni correction^79^ for multiple tests.

**Table 3:** Nonsynonymous substitution per nonsynonymous site (d_N_), synonymous substitutions per synonymous site (d_S_), ratio of d_N_/d_S_ (ω), and ratio of d_N_ over Kimura 2-parameter distance (K2) of COI between species of *Diadema* in the first exon (432 bp) of bindin. K2 for COI is based on data in Lessios *et al*.^33^.

Species 1	Species 2	d_N_^a^	d_S_^a^	ω		d_N_/K2_COI_
*D. africanum*	*D. antillarum*	0.004	0.011	0.350	^ns^	0.001
*D. africanum*	*D. paucispinum-*a	0.015	0.056	0.268	^ns^	0.004
*D. africanum*	*D. paucispinum-*a	0.015	0.054	0.277	^ns^	0.006
*D. africanum*	*D. paucispinum-*b	0.011	0.046	0.240	^ns^	0.003
*D. antillarum*	*D. paucispinum-*b	0.011	0.047	0.235	^ns^	0.004
*D. paucispinum-*a	*D. paucispinum-*b	0.007	0.040	0.175	^ns^	0.005
*D. africanum*	*D. savignyi*	0.011	0.054	0.204	^ns^	0.004
*D. antillarum*	*D. savignyi*	0.011	0.055	0.200	^ns^	0.004
*D. paucispinum-*a	*D. savignyi*	0.008	0.048	0.168	^ns^	0.003
*D. paucispinum-*b	*D. savignyi*	0.005	0.032	0.154	^ns^	0.002
*D. africanum*	*D. mexicanum*	0.011	0.037	0.296	^ns^	0.002
*D. antillarum*	*D. mexicanum*	0.010	0.040	0.248	^ns^	0.002
*D. paucispinum-*a	*D. mexicanum*	0.008	0.040	0.201	^ns^	0.002
*D. paucispinum-*b	*D. mexicanum*	0.005	0.047	0.107	^ns^	0.001
*D. savignyi*	*D. mexicanum*	0.005	0.052	0.097	^ns^	0.001
*D. africanum*	*D. palmeri*	0.050	0.090	0.553	^ns^	0.003
*D. antillarum*	*D. palmeri*	0.051	0.092	0.556	^ns^	0.003
*D. paucispinum-*a	*D. palmeri*	0.051	0.091	0.560	^ns^	0.003
*D. paucispinum-*b	*D. palmeri*	0.051	0.088	0.577	^ns^	0.003
*D. savignyi*	*D. palmeri*	0.052	0.098	0.531	^ns^	0.003
*D. mexicanum*	*D. palmeri*	0.052	0.099	0.527	^ns^	0.003
*D. africanum*	*D. clarki*	0.047	0.068	0.688	^ns^	0.004
*D. antillarum*	*D. clarki*	0.047	0.070	0.675	^ns^	0.004
*D. paucispinum-*a	*D. clarki*	0.052	0.070	0.745	^ns^	0.004
*D. paucispinum-*b	*D. clarki*	0.048	0.066	0.727	^ns^	0.004
*D. savignyi*	*D. clarki*	0.048	0.075	0.640	^ns^	0.004
*D. mexicanum*	*D. clarki*	0.046	0.076	0.607	^ns^	0.004
*D. palmeri*	*D. clarki*	0.045	0.060	0.748	^ns^	0.002
*D. africanum*	*D. setosum*-b	0.040	0.104	0.385	^ns^	0.002
*D. antillarum*	*D. setosum*-b	0.041	0.105	0.391	^ns^	0.002
*D. paucispinum-*a	*D. setosum*-b	0.046	0.108	0.427	^ns^	0.003
*D. paucispinum-*b	*D. setosum*-b	0.042	0.100	0.419	^ns^	0.002
*D. savignyi*	*D. setosum*-b	0.042	0.109	0.386	^ns^	0.003
*D. mexicanum*	*D. setosum*-b	0.042	0.108	0.387	^ns^	0.002
*D. palmeri*	*D. setosum*-b	0.057	0.075	0.759	^ns^	0.002
*D. clarki*	*D. setosum*-b	0.049	0.062	0.794	^ns^	0.002
*D. africanum*	*D. setosum-*a	0.044	0.116	0.379	^ns^	0.002
*D. antillarum*	*D. setosum-*a	0.044	0.118	0.372	^ns^	0.003
*D. paucispinum-*a	*D. setosum-*a	0.048	0.126	0.380	^ns^	0.003
*D. paucispinum-*b	*D. setosum-*a	0.044	0.117	0.375	^ns^	0.002
*D. savignyi*	*D. setosum-*a	0.044	0.125	0.351	^ns^	0.003
*D. mexicanum*	*D. setosum-*a	0.044	0.126	0.349	^ns^	0.003
*D. palmeri*	*D. setosum-*a	0.059	0.092	0.639	^ns^	0.003
*D. clarki*	*D. setosum-*a	0.052	0.079	0.660	^ns^	0.002
*D. setosum*-b	*D. setosum-*a	0.011	0.033	0.335	^ns^	0.001

^a ^Pamilio and Bianchi^58^ and Li^59^ method.

^ns^ ω not significantly different from 1 after sequential Bonferroni correction^79^ for multiple tests.

**Table 4:** Nonsynonymous substitution per nonsynonymous site (d_N_), synonymous substitutions per synonymous site (d_S_), ratio of d_N_/d_S_ (ω), and ratio of d_N_ over Kimura 2-parameter distance (K2) of COI between species of *Diadema* across the entire mature bindin gene of *Diadema* (1263 bp). K2 for COI is based on data in Lessios *et al*.^33^.

Species 1	Species 2	d_N_^a^	d_S_^a^	ω		d_N_/K2_COI_
*D. africanum*	*D. antillarum*	0.008	0.021	0.398	^c^	0.003
*D. africanum*	*D. paucispinum-*a	0.013	0.045	0.294	^c^	0.004
*D. antillarum*	*D. paucispinum-*a	0.013	0.041	0.304	^c^	0.005
*D. africanum*	*D. paucispinum-*b	0.012	0.042	0.296	^c^	0.003
*D. antillarum*	*D. paucispinum-*b	0.012	0.039	0.300	^c^	0.005
*D. paucispinum-*a	*D. paucispinum-*b	0.007	0.027	0.239	^c^	0.005
*D. africanum*	*D. savignyi*	0.013	0.044	0.298	^c^	0.005
*D. antillarum*	*D. savignyi*	0.012	0.041	0.303	^c^	0.005
*D. paucispinum-*a	*D. savignyi*	0.008	0.030	0.256	^c^	0.003
*D. paucispinum-*b	*D. savignyi*	0.006	0.022	0.272	^c^	0.002
*D. africanum*	*D. mexicanum*	0.013	0.034	0.373	^c^	0.002
*D. antillarum*	*D. mexicanum*	0.012	0.032	0.386	^c^	0.003
*D. paucispinum-*a	*D. mexicanum*	0.008	0.027	0.280	^c^	0.002
*D. paucispinum-*b	*D. mexicanum*	0.007	0.034	0.204	^c^	0.001
*D. savignyi*	*D. mexicanum*	0.007	0.033	0.208	^c^	0.002
*D. africanum*	*D. clarki*	0.049	0.078	0.619	^c^	0.004
*D. antillarum*	*D. clarki*	0.048	0.076	0.632	^c^	0.004
*D. paucispinum-*a	*D. clarki*	0.046	0.076	0.610	^c^	0.004
*D. paucispinum-*b	*D. clarki*	0.046	0.078	0.594	^c^	0.004
*D. savignyi*	*D. clarki*	0.047	0.080	0.586	^c^	0.004
*D. mexicanum*	*D. clarki*	0.047	0.078	0.603	^c^	0.004

^a ^Pamilio and Bianchi^58^ and Li^59^ method.

^c ^ω < 1 after sequential Bonferroni correction^79^ for multiple tests.

PAML codeml analyses that compared variation in ω among amino acid sites showed that models of positive selection were not statistically different from models of neutral evolution in either the first or the second exon of bindin (Tables 5 & 6). However, models that allowed for variation in ω values among clades fit the data significantly better than the null models which enforced a single ω value over all branches. The branch leading to *D. clarki* was estimated to have a very high ω (5.98 for the first exon and 6.6 for the second exon, Tables 5 and 6). Such an excess of replacement over silent substitutions is indicative of strong positive selection. The branch leading to *D. palmeri* also showed a value of ω higher than 1 in the first exon (1.48). We were not able to analyze the second exon of bindin in this species. In all other branches ω was considerably smaller than 1, indicating that selection was negative or that bindin evolved neutrally.

**Table 5:** A. Log-likelihood ratio tests comparing models of positive selection against null alternatives in the first exon of *Diadema* bindin. 2ΔL: Twice the difference of the log-likelihood of the models. k: number of ω classes. B. Models of variation of the ratio of the rates of amino acid replacement over silent substitutions (dN/dS = ω) in the first exon of bindin. pa: number of parameters. See text for explanation of models.

A			
Models compared	2ΔL	df	p
**Variation among sites**			
M1a vs. M2a	−0.0000	2	0.999
M7 vs. M8	−1.2520	2	0.535
M8 vs. M8a	−1.2521	1	0.272
**Variation among branches**			
MC vs. M2a_rel	−9.9747	4	0.041
MD (k = 3) vs M3	−11.2270	4	0.024
**B**			
**Model**	**Log Likelihood**	**pa**	**Parameter Estimates**
**Site-specific models**			
M1a (nearly neutral)	−1643.305	2	$\hat{p}$_0_ = 0.999
M2a (positive selection)	−1643.305	4	$\hat{p}$_0_ = 1.0, $\hat{p}$_1_ = 0.0, ($\hat{p}$_2_ = 0.0) $\hat{\omega }$_0_ = 0.521, $\hat{\omega }$_1_ = 1.0, $\hat{\omega }$_2_ = 1.0
M7 (beta)	−1642.654	2	$\hat{p}$ = 5.019, $\hat{q}$ = 4.580
M8 (beta&ω)	−1643.280	4	$\hat{p}$_0_ = 0.99999, ($\hat{p}$_1_ = 0.00001) $\hat{p}$ = 5.015, $\hat{q}$ = 4.577, $\hat{\omega }$ = 1.0
M8a (beta&ω = 1)	−1642.676	3	$\hat{p}$_0_ = 0.99999, ($\hat{p}$_1_ = 0.00001) $\hat{p}$ = 99.0, $\hat{q}$ = 91.067, $\hat{\omega }$ = 1.0
M2a_rel	−1642.678	4	$\hat{p}$ = 1.0, $\hat{p}$_1_ = 0.0, ($\hat{p}$_2_ = 0.0) $\hat{\omega }$_0_ = 0.521, $\hat{\omega }$_1_ = 1.0, $\hat{\omega }$_2_ = 0.0
M3 (discrete)	−1643.304	5	$\hat{p}$ = 0.0, $\hat{p}$_1_ = 0.0, ($\hat{p}$_2_ = 1.0) $\hat{\omega }$_0_ = 0.0, $\hat{\omega }$_1_ = 0.0, $\hat{\omega }$_2_ = 0.521
**Branch models**			
MC	−1637.691	8	Background: $\hat{p}$_0_ = 0.462, $\hat{p}$_1_ = 0.000, ($\hat{p}$_2_ = 0.538) $\hat{\omega }$_0_ = 0.540, $\hat{\omega }$_1_ = 1.0, $\hat{\omega }$_back_ = 0.313. Foreground: $\hat{\omega }$_for(palmeri)_ = 1.480, $\hat{\omega }$_for(clarki)_ = 5.981, $\hat{\omega }$_for(setosum-a)_ = 0.0001, $\hat{\omega }$_for(setosum-b)_ = 0.0001
MD (k = 3)	−1637.691	9	Background: $\hat{p}$_0_ = 0.232, $\hat{p}$_1_ = 0.230, ($\hat{p}$_2_ = 0.538) $\hat{\omega }$_0_ = 0.544, $\hat{\omega }$_1_ = 0.544, $\hat{\omega }$_back_ = 0.313. Foreground: $\hat{\omega }$_for(palmeri)_ = 1.480, $\hat{\omega }$_for(clarki)_ = 5.981, $\hat{\omega }$_for(setosum-a)_ = 0.0001, $\hat{\omega }$_for(setosum-b)_ = 0.0001

**Table 6:** A: Log-likelihood ratio tests comparing models of positive selection against null alternatives in the second exon of *Diadema* bindin. 2ΔL: Twice the difference of the log-likelihood of the models. k: number of ω classes. B: Models of variation of the ratio of the rates of amino acid replacement over silent substitutions (dN/dS = ω) in the second exon of bindin. pa: number of parameters. k: number of ω classes. See text for explanation of models.

A			
Models compared	2Δl	df	p
**Variable sites**			
M1a vs. M2a	−0.0000	2	1
M7 vs. M8	−0.4261	2	0.808
M8 vs. M8a	−0.0403	1	0.841
**Variable clades**			
MC vs. M2a_rel	−11.2629	4	0.004
MD (k = 3) vs M3	−11.4733	4	0.003
**B**			
**Model**	**l**	**pa**	**Parameter Estimates**
**Site-specific models**			
M1a (nearly neutral)	−2090.187	2	$\hat{p}$_0_ = 0.525
M2a (positive selection)	−2090.187	4	$\hat{p}$_0_ = 0.525, $\hat{p}$_1_ = 0.3, ($\hat{p}$_2_ = 0.176) $\hat{\omega }$_0_ = 0.0, $\hat{\omega }$_1_ = 1.0, $\hat{\omega }$_2_ = 1.0
M7 (beta)	−2090.187	2	$\hat{p}$ = 0.02, $\hat{q}$ = 0.023
M8 (beta&ω)	−2090.167	4	$\hat{p}$_0_ =0.997, ($\hat{p}$_1_ = 0.003) $\hat{p}$ = 0.005, $\hat{q}$ = 0.006, $\hat{\omega }$ = 5.09
M8a (beta&ω = 1)	−2090.187	3	$\hat{p}$_0_ = 0.525, ($\hat{p}$_1_ = 0.475) $\hat{p}$ = 0.005, $\hat{q}$ = 1.681, $\hat{\omega }$ = 1.0
M2a_rel	−2090.245	4	$\hat{p}$ = 0.520, $\hat{p}$_1_ = 0.110, ($\hat{p}$_2_ = 0.370) $\hat{\omega }$_0_ = 0.0, $\hat{\omega }$_1_ = 1.0, $\hat{\omega }$_2_ = 0.973
M3 (discrete)	−2090.345	5	$\hat{p}$ = 0.520, $\hat{p}$_1_ = 0.211, ($\hat{p}$_2_ = 0.268) $\hat{\omega }$_0_ = 0.0, $\hat{\omega }$_1_ = 0.979, $\hat{\omega }$_2_ = 0.979
**Clade models**			
MC	−2084.713	8	Background: $\hat{p}$_0_ = 0.517, $\hat{p}$_1_ = 0.360, ($\hat{p}$_2_ = 0.123) $\hat{\omega }$_0_ = 0.0, $\hat{\omega }$_1_ = 1.0, $\hat{\omega }$_back_ = 0.16. Foreground: $\hat{\omega }$_for(*clarki*)_ = 6.608.
MD (k = 3)	−2084.608	9	Background: $\hat{p}$_0_ = 0.53, $\hat{p}$_1_ = 0.34, ($\hat{p}$_2_ = 0.13) $\hat{\omega }$_0_ = 0.0, $\hat{\omega }$_1_ = 1.114, $\hat{\omega }$_back_ = 0.17. Foreground: $\hat{\omega }$_for(*clarki*)_ = 6.561.

**Figure 3:**
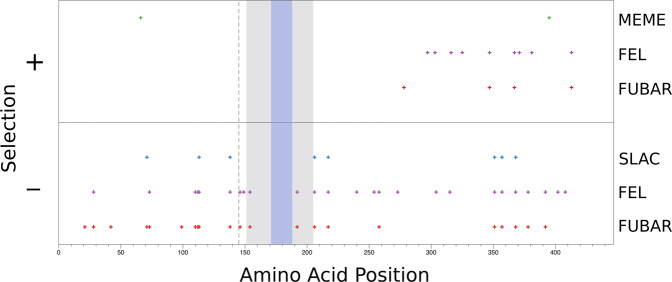
Codons under negative (below horizontal line) and positive (above horizontal line) selection in the entire bindin molecule of *Diadema* based on tests FEL, SLAC, FUBAR and MEME of program HyPhy^61^ applied to bindin alleles of all species of *Diadema*. Amino acid positions along the length of the mature protein (Supplementary Fig. S1 & S2) are marked on the horizontal axis. The dotted vertical line marks the position of the intron. The lightly shaded region indicates the position of the conserved core of bindin, and the dark shading indicates the highly conserved B18 region^100^ of the core.

Maximum likelihood and Bayesian analyses (FEL, SLAC, and FUBAR) were applied to the first exon of bindin in all *Diadema* species, and to the entire length of the mature bindin molecule for the species in which the second exon could be sequenced. They identified specific sites subject to positive or negative selection. The three methods generally converged on the same negatively selected sites, although there were 15 sites that were only identified by a single method (Fig. 3).

Only three sites were determined to be under positive selection by at least two methods. SLAC failed to detect any sites under positive selection. FEL was the most liberal, identifying 6 sites under positive selection. FUBAR identified 4 sites as being under positive selection, but only one of these sites, 278, was not also identified by FEL (Table S3). Only MEME identified any sites in the first exon as experiencing positive selection. Site 66 changed from Leucine (L) to Histidine (H) on the branch leading to all bindin sequences of *D. palmeri*. The same site changed from Leucine to Glutamine on the branch leading to a cluster of alleles from *D. paucispinum* and *D. savignyi*. All other sites under positive selection were on the second exon of bindin (Fig. 3). When the three positively selected sites in which FUBAR and FEL agreed were mapped on the bindin gene genealogy using parsimony, they were found to have changed multiple times across the tree (Fig. 2). Close to the base of the tree, amino acid site 367 changed from an Arginine (R) to a Glycine (G) and site 413 changed from Glycine (G) to Alanine (A). Both of these sites changed again at more terminal branches. Amino acid site 347 changed from an Isoleucine (I) to a Valine (V) at the branch leading to the majority of the *D. africanum* and *D. antillarum* sequences but experienced at least one reversal at a terminal node containing a bindin sequence of *D. antillarum*.

MEME is a method much different than the previous three, designed to detect “episodic” positive selection occurring on specific branches, selection that can be masked by purifying selection elsewhere in the tree^74^. MEME identified two sites, not identified by any other method. The first, a change from Lysine (L) to Glutamine (Q) at amino acid site 66 mapped with parsimony to a node which unites all of the sequences recovered from *D. paucispinum*-a, plus some from *D. paucispinum*-b and *D. savignyi*. This same site experienced a change from Lysine (L) to Histidine (H) at the node which unites all sequences of *D. palmeri* (Fig. 1). The second site, a change from Proline (P) to Tyrosine (Y) at amino acid position 395, represents a change at a terminal node containing a sequence from *D. paucispinum*-b.

## Discussion

### Evolution of *Diadema* bindin

With the exception of the branch leading to *Diadema clarki* (as indicated by PAML), there is very little evidence of positive selection in the evolution of bindin of *Diadema*. FUBAR, FEL, SLAC, and MEME disagreed with each other regarding sites considered to be evolving under positive selection, but they consistently suggested that most sites are either under negative selection or evolving neutrally. Although (as expected for nuclear genes evolving neutrally or under purifying selection^80,81^) the bindin trees (Fig. 1 & 2) show much lower resolution, they are compatible with the mitochondrial tree^33^, suggesting that divergence in bindin is mostly a function of time.

The absence of positive selection on bindin of *Diadema* is also reflected in the rate of evolution of the molecule in this genus. In the entire molecule, there is only one fixed amino acid difference between *D. mexicanum* and *D. antillarum*, separated for a minimum of 2.5 million years^33^. In the first exon, there are only five fixed amino acid differences between the clade leading to *D. setosum* and the clade leading to the rest of the species. These two major clades separated 12–14 million years ago. By comparison, the full mature bindin between two species of *Echinometra* separated for 1.5 million years have seven fixed amino acid differences^13^. If the rate of adaptive divergence of *Diadema* bindin between species is measured as the mean ratio of amino acid replacement mutations per replacement site between species in bindin divided by divergence in Cytochrome Oxidase I (as a proxy of divergence time)^10^, this ratio (d_N(ex1)_/K2P_(COI)_ = 0.285 for the first exon, d_N(ex2)_/K2P_(COI)_ = 0.352 for the second exon, and d_N(bindin)_/K2P_(COI)_ = 0.349 for the entire molecule) indicates that bindin evolution in this genus has been very slow (Tables 3 and 4). It is as slow as the rate of bindin divergence of two species of *Pseudoboletia* that hybridize extensively^21^ and four times as slow as that of the bindin of *Echinometra*, in which bindin evolves under positive selection (see Table 14.1 in ref. ^10^). Indo-Pacific species of *Diadema* also hybridize^41^, (albeit at a much lower rate than those of *Pseudoboletia*), and this may explain the presence of shared bindin alleles in *D. savignyi* and *D. paucispinum*-b. The most likely explanation, however, is that the slow rate of bindin evolution, coupled with the recent splitting of these species, has yet to sort out polymorphisms. Thus, bindin in *Diadema* evolves slowly under purifying selection, with little adaptive divergence in all species except, perhaps, for *D. clarki*.

Gametic recognition proteins are held to evolve rapidly under positive selection^3,7,8^ and to contribute to reproductive isolation, but bindin in more than half of the sea urchin genera studied to date appears to evolve mostly under purifying selection, as predicted by Kimura’s^82^ neutral theory. Why should this be the case for bindin evolution in *Diadema*? And why should the bindin of *D. clarki* evolve in a different manner than that of the bindin of all other species of *Diadema*? An early, attractive hypothesis was that the selective force acting on bindin was avoidance of hybridization^13^. This hypothesis appeared to hold in a general sense, because positive selection was detected in genera, such as *Echinometra*, that had sympatric species^12,13^, but was absent in genera, such as *Tripneustes*, in which all species were allopatric^22^. *Diadema*, with four sympatric species in the Western Pacific and single species in the eastern Pacific and on each of the two sides of the Atlantic, would have appeared to be ideal for testing the reinforcement hypothesis. The results, however, are mixed. *D. clarki*, sympatric with *D. setosum* and *D. savignyi*, does have bindin that evolves under positive selection. *D. setosum* and *D. savignyi*, on the other hand, do not. Neither does *D. paucispinum*, the range of which may overlap with *D. savignyi*^33,41^.

The lack of fast bindin evolution in all but one species of *Diadema* could be due to the presence of other isolating barriers. As Coyne and Orr^83^ have stressed, selection for prezygotic reproductive isolation will be strongest on barriers that act early in the sequence of species recognition between individuals. In sea urchins, possible prezygotic barriers to interspecific mating, arranged in the order in which they would act, are (1) habitat separation, (2) allochronic spawning, (3) differences in chemical attraction of egg and sperm, (4) lack of activation of the acrosome reaction of the sperm by the egg jelly, and (5) prevention of penetration of the egg and fusion of the vitelline layer with the acrosome process^26^. Thus, selection on bindin would be relaxed if one of the earlier steps blocks interspecific fertilization before egg and sperm can come in intimate contact. Our knowledge of *Diadema* ecology and egg-sperm interactions is far from complete, but it does provide clues on some of the steps that could affect species recognition before bindin comes into play.

Although there is considerable confusion in the literature, arising from the lack of reliable diagnostic morphological characters between species of *Diadema*^84^, the ranges of *D. setosum* and *D. savignyi* overlap west of Tonga^35^, but *D. savignyi* in the Central Pacific potentially coexists only with *D. paucispinum*^33^. On a much finer scale, *D. setosum* and *D. savignyi* show some differences in the microhabitat they occupy^85^, but they can often be found in mixed aggregations^44,84^. *D. clarki* is known from only part of the range of *D. setosum* and *D. savignyi* in Japan^38^, Indonesia^39^, and the Marshal Islands^33^, but this species had been synonymized by Mortensen^86^ with *D. setosum* after its original description by Ikeda^87^ and was only resurrected in 2014 by Chow *et al*.^37^. It is, thus, likely to be much more widespread. It does not appear to occupy a separate habitat than *D. setosum* and *D. savignyi*^38^. Thus, spatial segregation of species of *Diadema* with overlapping geographical ranges is not likely to be an effective barrier to fertilization opportunities.

Allochronic spawning may be the reason that *D. setosum*-a and *D. savignyi* experience no selection for adaptive divergence in bindin. *D. savignyi* spawns at the full moon^48,88^. *D. setosum* spawns at the new moon in most locations in which its reproductive cycle has been studied^48^, although a number of individuals continue to have gametes during the rest of the lunar cycle^88^, and its reproduction may be geographically variable^89^. These non-overlapping spawning cycles do not appear to have evolved by reinforcement, because they are also present in the eastern Pacific *D. mexicanum* (spawning at full moon) and the western Atlantic *D. antillarum*^45^ (spawning at new moon, as does *D. africanum* (J.C Hernandez pers.com)) Nevertheless, when present, they would obviate the evolution of barriers against hybridization at the level of gamete interactions. Binks *et al*.^25^ have attributed the lack of divergence at bindin in two subspecies of *Heliocidaris erythrogramma* in western Australia to the asynchrony of their reproductive cycles. The monthly reproductive cycles of *D. clarki* and *D. paucispinum* have not been studied. The question of whether bindin is under selection in *D. clarki* because the reproductive cycle of this species overlaps with that of one of the other two species of *Diadema*, with which it is sympatric, remains open.

Another open question concerns molecules that precede bindin in interaction between gametes^90^. They may also shield bindin from selection against hybridization. Speract and its receptor, which are involved in sperm activation and in attraction between egg and sperm, evolve under negative selection in *Diadema*^91^ and are, therefore, not likely to be involved in protecting against heterospecific fertilizations. Nothing is known about the evolution of the Sea Urchin Receptor for Egg Jelly (suREJ) from any sea urchin genus other than *Strongylocentrotus*. In *Strongylocentrotus* it evolves under positive selection^92^, but this has not prevented positive selection from also acting on the bindin of this genus^15^.

In a series of papers, Levitan and colleagues have documented that the danger of polyspermy in *Strongylocentrotus* sets the stage for differential selection on bindin, depending on the fertilization environment^27,29,31,93,94^. When sperm is limited, bindin alleles that are most compatible with alleles of the egg receptor are most likely to be successful. When an excess of sperm surrounds the eggs, compatible alleles result in fatal polyspermic fertilizations, but less compatible alleles are more likely to produce viable embryos. Thus, under limited sperm concentrations there should be purifying selection for high affinity gametes, whereas under high sperm concentrations sexual conflict should predominate. According to Levitan and colleagues^29,94^, shifting population densities through time and negative frequency-dependent selection should create distinct compatibility groups of bindin and receptor alleles, which could eventually lead to either balanced polymorphism or to reproductive isolation. If this pattern were generally applicable to sea urchin fertilization, the prediction would be that bindin in species that spawn in intraspecific synchrony in dense clusters should show evidence of positive selection, whereas bindin of species in which males and females are separated at times of spawning should be under purifying selection, as gametic compatibility would be at a premium^27^. Species of *Diadema* as a rule are distributed in patches with high point population densities, and they form spawning aggregations (reviewed in ref. ^84^). Even *D. antillarum*, which suffered mass mortality^95^ from which it has been slow to recover almost forty years later^96^, had high effective population sizes for at least 100,000 years^97^. How the reduced population densities would affect bindin gene frequencies if they were to persist for centuries or millennia could be modeled, but the four decades since mass mortality is too short a time to be expected to have produced notable effects. Two decades were not sufficient to reduce variation in mitochondrial DNA^97^. Episodic selection resulting in balanced polymorphisms (and thus reducing the trend towards overall positive selection) is what MEME is designed to detect. Though the power of this approach is still an open question, in *Diadema*, it has detected very few sites that have experienced positive selection at some point in their evolution. Thus, most spawning in *Diadema* likely results in high sperm densities, yet *Diadema* bindin shows little evidence of positive selection. It may be that the very small eggs of this genus, with a diameter of 68 µm^98^, present a small target for the sperm^99^ and thus reduce the danger of polyspermy. Or it may simply be that processes documented as acting on the bindin of *Strongylocentrotus* do not apply to other genera.

## Conclusion

Despite being a gamete recognition protein, the bindin of *Diadema* evolves slowly under purifying selection, like the bindin in four other sea urchin genera. All the genera in which fast bindin evolution has been found are members of the order Echinoida. *Diadema* joins *Arbacia* as a genus that does not belong to this order and also shows little evidence of selection on its bindin. However, whether evolution in bindin will be fast or slow does not appear to be phylogenetically determined, as the bindin of *Lytechinus*, *Tripneustes*, and *Pseudoboletia*, also members of the Echinoida, evolves slowly. The reasons as to why bindin evolves fast in some genera and not in others remain obscure; however, bindin, unlike other gamete recognition proteins, has now been studied in nine genera in three orders of an entire class or organisms. The diversity of its modes of evolution may simply be a reflection of the extent of organismal diversity that has received attention, in contrast to other gamete recognition proteins, studies of which have, as a rule, focused on a single genus.

## Supplementary information

Supplementary information.

## Data Availability

All data generated and analyzed for this study have been deposited in GenBank, Accession #s MT365802-MT365868 and MT375187-MT375188.
