# Supplementary material for: Slow evolution under purifying selection in the gamete recognition protein bindin of the sea urchin Diadema
Source: Sci Rep. 2020 Jun 17;10:9834. doi: 10.1038/s41598-020-66390-2 (PMC7299941; doi:10.1038/s41598-020-66390-2)
Supplement: Supplementary file 1 — Supplementary information. [file 41598_2020_66390_MOESM1_ESM.pdf]

Slow evolution under purifying selection in the gamete recognition protein bindin of the sea urchin *Diadema*

L. B. Geyer

K. S. Zigler

S. Tiozzo

H. A. Lessios

Supplementary Data

Table S1. Amplification and sequencing primers designed for the mature bindin gene in *Diadema*.

Primer name	Primer Sequence	tm	Used in:	orientation
DA5A	GATTTCTTTATGGGACATCGCAA	51.7°C	amplification	forward
DA3R2	CATTCGGAACACATCACTCTCC	54.8°C	amplification	reverse
DAIR	TCCGCACTGATGGTATCGTC	53.8°C	amplification	reverse
MESDQPP	ATGGAGTCTGATCAGCCCCC	55.9°C	sequencing	forward
QVLTAMQE	CAAGTTTTGACCGCTATGCAGGA	55.3°C	sequencing	forward
LNAGGGE	TCTCAATGCAGGGGGTGGAGAG	58.6°C	sequencing	forward
NSQEEPSD	AATAGTCAGGAAGAACCGAGCG	54.8°C	sequencing	forward
EEVGQAS	CGAGGAAGGTGTTCAAGCTTCCC	58.8°C	sequencing	forward
DIABINrev7	CTTTGTCGCACCGAGTACAGC	56.3°C	sequencing	reverse
DJINR	CTGGCTTTCTTGATCGAAGTC	52.4°C	sequencing	reverse

Table S2. Probablities of negative selection for amino acid sites identified by maximum likelihood (FEL and SLAC) and Bayesian (FUBAR) methods. Shading indicates sites for which probabilities are significant at the 0.05 level.

Amino Acid Site	FEL [a]	SLAC [a]	FUBAR [b]
21	0.061	0.1111	0.938
28	0.048	0.1111	0.935
42	0.254	0.2580	0.914
71	0.064	0.0457	0.941
73	0.044	0.1134	0.942
99	0.141	0.1111	0.961
110	0.046	0.1111	0.934
112	0.046	0.1111	0.936
113	0.002	0.0123	0.999
138	0.004	0.0071	0.995
146	0.028	0.1457	0.986
149	0.043	0.1121	0.874
154	0.042	0.1128	0.943
192	0.030	0.1111	0.963
206	0.013	0.0498	0.982
217	0.000	0.0006	1.000
240	0.016	0.2582	0.818
252	0.011	0.4415	0.594
258	0.002	0.0547	0.978
273	0.010	0.2612	0.837
304	0.047	0.3335	0.740
315	0.000	0.3535	0.633
351	0.000	0.0068	0.997
357	0.000	0.0072	0.992
368	0.000	0.0113	0.994
378	0.000	0.1111	0.945
392	0.000	0.0578	0.991
402	0.011	0.3516	0.749
408	0.016	0.3333	0.828

[a] p values of the Likelihood Ratio Test

[b] Bayesian posterior probability that $d_N<d_S$

Table S3. Probablities of positive selection for amino acid sites identified by maximum likelihood (MEME, FEL and SLAC) and Bayesian (FUBAR) methods. Shading indicates sites for which probabilities are significant at the 0.05 level.

Amino Acid Site	MEME [a]	FEL [a]	SLAC [a]	FUBAR [b]
66	0.01	0.624	0.671	0.646
278	0.09	0.150	0.537	0.902
297	0.32	0.011	0.649	0.781
303	0.23	0.001	0.667	0.689
316	0.16	0.000	0.550	0.833
325	0.24	0.010	0.616	0.804
347	0.11	0.015	0.274	0.960
367	0.11	0.002	0.296	0.929
371	0.18	0.034	0.446	0.817
381	0.19	0.003	0.442	0.824
395	0.00	0.150	0.445	0.838
413	0.19	0.001	0.461	0.915

[a] p values of the Likelihood Ratio Test

[b] Bayesian posterior probability that $d_N > d_S$

```
                    10        20        30        40        50        60        70        80        90       100       110       120       130       140
                     |         |         |         |         |         |         |         |         |         |         |         |         |         |
D.afr_1.1   YNPTGNVGRAGAQQGGGTRAAYPPAQSGRPNYYDPRANNPQPAYAGREYGA-PGARQQQYMENPSLPSGRANGAAPGPRQGAPSGHIPGPAYGPSYGPAPAGPVIPPAEGAGGDFDSVSRTMESDQPLHEYSLSSSQDDTISAE
D.afr_1.2   ...................................................-...........................................................................................D
D.afr_2.1   ...................................................-............................................................................................
D.afr_3.1   ...................................................-...........................................................................................?
D.afr_3.2   ................................................?..-............................................................................................
D.afr_4.1   ...................................................-...........................................................................................D
D.afr_4.2   ...................................................-............................................................................................
D.afr_5.1   ...................................................-............................................................................................
D.afr_5.2   ...................................................-........V...................................................................................
D.afr_6.1   ...................................................-............................................................................................
D.afr_7.1   ...................................................-............................................................................................
D.afr_8.1   ...................................................-............................................................................................
D.ant_1.1   .................................G.................-...H................................................................................????????
D.ant_2.1   ...................................................-...................?.............................?..........................................
D.ant_2.2   ...................................................-.........................L.................................................................D
D.ant_3.1   ...................................................-............................................................................................
D.ant_4.1   ...................................................-............................................................................................
D.ant_4.2   ...................................................-............................................................................................
D.ant_5.1   .................................G.................-...H.....................L.........................G.......................................D
D.ant_5.2   .................................G.................-...H..........................?............................................................D
D.ant_6.1   ...................................................-............................................................................................
D.ant_6.2   ...................................................-...........................................C................................................
D.ant_7.1   ...................................................-............................................................................................
D.ant_8.1   ...................................................-.....................................?......................................................
D.ant_8.2   ...................................................-............................................................................................
D.ant_9.1   ...................................................-............................................................................................
D.ant_10.1  ...................................................-............................................................................................
D.ant_11.1  ...................................................-.................H.......................................................R.................D
D.ant_11.2  ...................................................-............................................................................................
D.ant_12.2  .............H.....................................-............................................................................................
D.ant_12.1  ...................................................-............................................................................................
D.ant_13.1  ...................................................-............................................................................................
D.ant_14.1  .................................G.................-...H.................................................................................???????
D.p-a_1.1   .................................G.......Q.........-...H.........Q.............................................................................D
D.p-a_2.1   .................................G.......Q.........-...H.........Q.............................................................................D
D.p-a_3.1   .................................G.................-...H.........Q.............................................................................D
D.p-a_4.1   .................................G.................-...H.........Q.............................................................................D
D.p-a_5.1   .................................G.................-...H.........Q.............................................................................D
D.p-a_6.1   .................................G.................-...H.........Q.............................................................................D
D.p-a_6.2   .................................G.................-...H.........Q........................................................................N....D
D.p-a_7.1   .................................G.................-...H.........Q.............................................................................D
D.p-a_8.1   .................................G.................-...H.........Q.......................................................................???????
D.p-a_9.1   .................................G.................-...H.........Q.......................................................................???????
D.p-b_1.1   .................................G.................-...H.......................................................................................D
D.p-b_1.2   .................................G.................-...H...........................A...........................................................D
D.p-b_2.1   .................................G.................-...H.......................................................................................D
D.p-b_3.1   .................................G.................-...H.......................................................................................D
D.p-b_4.1   ...P.............................G.................-...H.......................................................................................D
D.p-b_4.2   .................................G.................-...H.................?......................................?..............................D
D.p-b_5.1   .................................G.................-...H.........Q......................................V......................................D
D.p-b_5.2   ..........?......................G.................-...H.........Q.............................................................................D
D.p-b_6.1   ...................................................-...........................................................................................D
D.p-b_6.2   .................................G.................-...H....................................................................Y..................D
D.p-b_7.1   .................................G.................-...H................................................................................????????
D.p-b_8.1   .................................G.................-...H.................................................................................???????
D.p-b_9.1   .................................G.................-...H.................V.....................................................................D
D.p-b_9.2   .................................G.................-...H.................V.....................................................................D
D.p-b_10.1  .................................G.................-...H.......................................................................................D
D.sav_1.1   .................................G.................-...H..........................................V............................................D
D.sav_1.2   ............R....................G.................-...H.......................................................................................D
D.sav_2.1   .................................G.................-...H...........................A...........................................................D
D.sav_2.2   .................................G.................-...H...........................A...........................................................D
D.sav_3.1   .................................G.................-...H.......................................................................................D
D.sav_3.2   .................................G.................-...H.......................................................................................D
D.sav_4.1   .................................G.................-...H.......................................................................................D
D.sav_4.2   .................................G.................-...H..........?.....?..............................................................?.......D
D.sav_5.1   .................................G.................-...H.......................................................................................D
D.sav_5.2   .................................G.................-...H.......................................................................................D
D.sav_6.1   .................................G.................-...H.......................................................................................D
D.sav_6.2   .................................G.................-...H...............................................................?.......................D
D.sav_7.1   .................................G.................-...H.................................................................................???????
D.sav_8.1   .................................G.................-...H.................................................................................???????
D.sav_9.1   .................................G.................-...H.............?.........................................................................D
D.sav_9.2   .................................G.................-...H.......................................................................................D
D.sav_10.1  .........S.....?...G...............................-.......................S...................................................................D
D.sav_10.2  .........S.....S...G...............................-.......................S...................................................................D
D.sav_12.1  .................................G.................-...H.........Q.............................................................................D
D.mex_1.1   .................................G.................-...H.......................................................................................D
D.mex_2.1   .................................G.................-...H.......................................................................................D
D.mex_3.1   .........S.........................................P....................................................................................E......D
D.mex_4.1   .................................G.................-...H..............V........................................................................D
D.mex_4.2   .................................G.................-...H...........P..V........................................................................D
D.mex_5.1   .................................G.................-...H.......................................................................................D
D.mex_6.1   .................................G.................-...H...........................................................................?...........D
D.mex_7.1   .................................G.................-...H.......................................................................................D
D.mex_8.1   .................................G.................-...H.......................................................................................D
D.mex_8.2   .................................G.................-...H.......................................................................................D
D.pal_1.1   ............................P....G...-.............-....P........H.................P......................Q.........I..Q.A...E.PN...F....???????
D.pal_3.1   ................S...........P....G...-.............-....P........H.................P......................Q.........I..Q.A...E.PN...F..........?
D.pal_4.1   ............................P....G...-.............-....P........H.................P......................Q.........I..Q.A...E.PN...F..........?
D.cla_1.1   .............H..............P....G...-..P.........V-....P..........P..G............P.....................................V.....P....F...L......D
D.cla_2.1   .............H........S.....P....G...-..P.........V-....P..........P..G............P.....................................V.....P....F...L......D
D.cla_3.1   .............H..............P....G...-..P..........-....P..........P..G............P.....................................V.....P...?F...L......D
D.cla_3.2   .............H........S.....P....G...-..P.........V-....P..........P..G............P.....................................V.....P....F...L......D
D.cla_4.1   .............H..............P....G...-..P..........-....P..........P..G............P.....................................V.....P....F...L???????
D.cla_5.1   .............H..............P....G...-..P.........V-....P..........P..G............P................S....................V.....P....F...L......?
D.cla_5.2   .............H..............P....G...-D.P..........-....P..........P..G............P.....................................V.....P....F...L......?
D.cla_6.1   .............H...S..........P....G...-..P..........-....P..........P..G............P.....................................V.....P....F...L.......
D.cla_6.1   .............H...S..........P....G...-..P..........-....P..........P..G............P.....................................V.....P....F...L.......
D.cla_7.1   .............H..............P....G...-..P.........V-....P..........P..G............P.....................................V.....P....F...L......?
D.cla_8.1   .............H........S.....P....G...-..P.........V-....P..........P..G............P.....................................V.....P....F...L???????
D.cla_9.1   .............H..............P....G...-..P.........V-....P..........P..G............P.....................................V.....P....F...L......?
D.cla_10.1  .............H..............P....G...-..P.........V-....P..........P..G............P............?........................V.....P....F...L......?
D.cla_11.1  .............H..............P....G...-..P.........V-....P..........P..G............P............?........................V.....P....F...L......?
D.s-a_1.1   ...........V...........S....P....G...-........H....-.....H.........................P..M............................E...........PQ...F..........?
D.s-a_1.2   ............................P....G...-........H....-.....H.........................P..M............................E...........PQ...F..........?
D.s-a_2.1   ...........V...........S....P....G...-........H....-.....H.........................P..M............................E...........PQ...F..........?
D.s-a_4.1   ...........V...........S....P....G...-........H....-.....H.........................P..M............................E...........PQ...F..........?
D.s-a_5.1   ...........V...........S....P....G...-........H....-.....H.........................P..M............................E...........PQ...F..........?
D.s-a_6.1   ...........V...........S....P....G...-........H....-.....H.........................P...............................E...........PQ...F....???????
D.s-a_7.1   ...........V...........S....?....G...-........H....-.....H.........................P..M............................E...........PQ...F...........
D.s-a_8.1   ...........V...........S....P....G...-........H....-.....H.........................P..M............................E...........PQ...F..........?
D.s-a_8.2   ...........V...........S....S....G...-........H....-.....H.........................P...............................E...........PQ...F..........?
D.s-a_9.1   ...........V...........S....P....G...-........H....-.....H........................?P..M............................E...........PQ...F..........?
D.s-a_10.1  ...........V...........S....P....G...-........H....-.....H.........................P..M............................E...........PQ...F..........?
D.s-a_11.1  ...........V...........S....P....G...-........H....-.....H.............S...........P..M............................E...........PQ...F....???????
D.s-b_1.1   ...........V...........S....P....G...-........H....-...............................P...............................Q...........PQ...F..........?
D.s-b_2.1   ...........V...........S....P....G...-........H....-...............................P...............................Q...........PQ...F...........
D.s-b_3.1   ...........V...........S....P....G...-........H....-...............................P...............................Q...........PQ...F..........?
D.s-b_4.1   ...........V...........S....P....G...-........H....-...............................P...............................Q...........PQ...F....???????
D.s-b_5.1   ...........V...........S....P....G...-........H....-...............................P...............................Q...........PQG..F...????????
D.s-b_6.1   ...........V...........S....P....G...-........H....-...............................P...............................Q...........PQ...F..........?
D.s-b_7.1   ...........V...........S....P....G...-........H....-...............................P...............................Q...........PQ...F..........?
D.s-b_8.1   ...........V...........S....P....G...-........H....-...............................P...............................Q...........PQ...F...........
D.s-b_9.1   ...........V...........S....P....G...-........H....-...............................P...............................Q...........PQ...F..........?
D.s-b_10.1  ...........V...........S....P....G...-........H....-...............................P..........................P....Q...........PQ...F..........?
```

Figure S1. Amino acid alignment of alleles of the bindin first exon of Diadema species. See Fig. 1 for explanation of codes

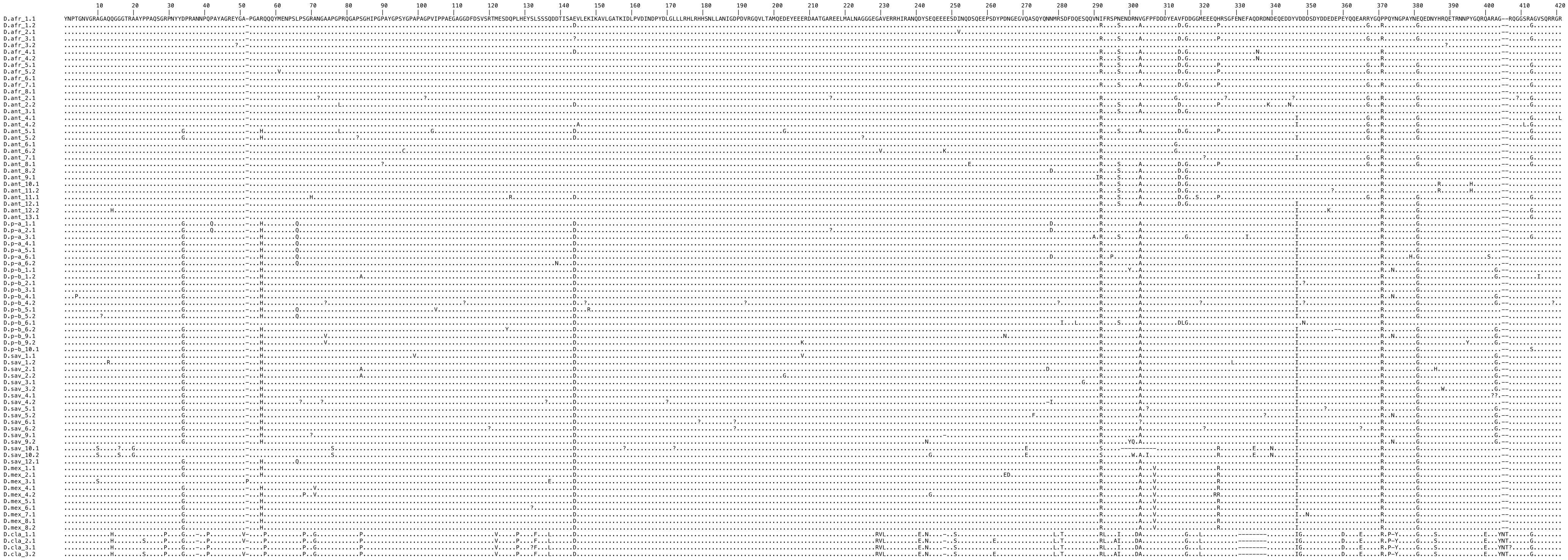

Fig S2. Amino acid alignment of the entire bindin molecule of Diadema species. See Fig. 1 for explanation of codes
